# GlassesViewer: Open-source software for viewing and analyzing data from the Tobii Pro Glasses 2 eye tracker

**DOI:** 10.3758/s13428-019-01314-1

**Published:** 2020-01-02

**Authors:** Diederick C. Niehorster, Roy S. Hessels, Jeroen S. Benjamins

**Affiliations:** 1 Lund University Humanities Laboratory and Department of Psychology, Lund University, Lund, Sweden; 2 Experimental Psychology, Helmholtz Institute and Developmental Psychology, Utrecht University, Utrecht, The Netherlands; 3 Experimental Psychology, Helmholtz Institute and Social, Health and Organisational Psychology, Utrecht University, Utrecht, The Netherlands

**Keywords:** Head-mounted eye tracking, Wearable eye tracking, Mobile eye tracking, Eye movements, Data analysis, Event classification

## Abstract

We present GlassesViewer, open-source software for viewing and analyzing eye-tracking data of the Tobii Pro Glasses 2 head-mounted eye tracker as well as the scene and eye videos and other data streams (pupil size, gyroscope, accelerometer, and TTL input) that this headset can record. The software provides the following functionality written in MATLAB: (1) a graphical interface for navigating the study- and recording structure produced by the Tobii Glasses 2; (2) functionality to unpack, parse, and synchronize the various data and video streams comprising a Glasses 2 recording; and (3) a graphical interface for viewing the Glasses 2’s gaze direction, pupil size, gyroscope and accelerometer time-series data, along with the recorded scene and eye camera videos. In this latter interface, segments of data can furthermore be labeled through user-provided event classification algorithms or by means of manual annotation. Lastly, the toolbox provides integration with the GazeCode tool by Benjamins et al. (2018), enabling a completely open-source workflow for analyzing Tobii Pro Glasses 2 recordings.

## Introduction

In the past several decades, mobile (head-worn) eye tracking has become a popular research method that has found widespread use across a range of fields. The opportunity afforded by head-worn eye-tracking setups to acquire data on the visual behavior of participants who freely move around has enabled researchers to conduct studies in fields such as vision science (Land, 1992; Land & Lee, 1994; Ballard et al., 1995; Pelz & Canosa, 2001; Matthis et al., 2018), social interaction between adults (Ho et al., 2015; Rogers et al., 2018; Macdonald & Tatler, 2018; Rogers et al., 2019) or children and their parents (Yu & Smith, 2017; Suarez-Rivera et al., 2019), usability (Masood & Thigambaram, 2015; Bergstrom & Schall, 2014), marketing (Harwood & Jones, 2014), decision making (Gidlöf et al., 2013; Gidlöf et al., 2017), surgery (Dik et al., 2016; Harrison et al., 2016), navigation and wayfinding (Kiefer et al., 2014; Koletsis et al., 2017) and education (McIntyre et al., 2017; McIntyre & Foulsham, 2018).

Head-worn eye trackers typically consist of some form of headset or glasses on which multiple cameras are mounted. First, there is a scene camera that is pointed forward and films the world in front of the participant. Second, there are one or more cameras that film one or both eyes of the participant. The images from one or more eye cameras are processed by firmware or software—the headset and the gaze processing code together form an eye-tracking setup. The typical output of a head-worn eye-tracking setup consists of the video of the scene camera along with gaze direction, usually reported in the video frame of the scene camera. The Tobii Pro Glasses 2, a system that records binocular gaze direction, furthermore provides pupil size, the 3D orientation of each eye ball in a coordinate system fixed to the headset, and gyroscope and accelerometer data indicating movement of the headset.

Analysis of head-worn eye-tracking data often happens in multiple steps. First, event classification (the labeling of parts of, e.g., the eye-tracking data as, e.g., “fixations” and “saccades”, see Hessels et al.,, 2018) is commonly performed to extract episodes of the recording for further analysis. Important to note here is that only few event classification algorithms are available that are developed for head-worn eye tracker signals (Hooge & Camps, 2013; Hessels et al., in press; Larsson & et al. 2016; Kothari et al., 2019), and that none of these appear to be implemented in commercial software for head-worn eye-tracking data analysis.

In many fields, such as psychology, researchers using head-worn eye trackers are predominantly not interested in how a participant’s eyes move in their head, but instead in questions such as which objects in the world a person looked at, in what order, and how long each object was looked at (see Hessels et al.,, 2018, for a discussion of coordinate systems in which gaze direction can be measured). As such, after event classification, a common step is to use the classified fixations to determine what objects an observer looked at. This is however not straightforward for most head-mounted eye-tracking research. Unlike in screen-based eye tracking where the experimenter often has control over the stimulus and knows what object was presented where and when, the positions of objects in the scene video of a head-worn eye tracker are usually unknown. Since the scene video provided by the head-worn eye tracker is recorded from the perspective of a moving observer, objects of interest continuously change position when the observer moves, or even disappear from view altogether. Analysis of head-mounted eye-tracking data therefore requires mapping the gaze direction provided by the eye tracker in the reference frame of the scene video to specific objects in the world. Unless the position and orientation of the headset and the objects of interest in the world are known, or if the location of objects in the scene video can be recovered by automatic means (e.g., Brône et al.,, 2011), mapping gaze to the object in the world is often carried out manually. We will refer to this as *manual mapping* in this paper. While some researchers perform manual mapping for individual video frames or gaze samples (e.g., Land et al.,, 1999; Gidlöf et al.,, 2013; Gidlöf et al.,, 2017), frequently researchers first obtain episodes in the recording where gaze is fixed on an object in the world (often called fixation classification or event classification), and then code these episodes one at a time (see, e.g., Hessels et al.,, in press).

The Tobii Pro Glasses 2 is a recent head-worn eye tracker that is commonly used across a range of studies (e.g., Harrison et al.,, 2016; Topolšek et al.,, 2016; Koletsis et al.,, 2017; Rogers et al.,, 2018; Raptis et al.,, 2018). However, analysis of the recordings is currently limited to Tobii Pro Lab, a software package sold by the manufacturer of this eye tracker, and other closed commercial software packages such as offered by iMotions. Tobii Pro Lab aims to provide the analysis options that a majority of Tobii’s academic and industry clients want. For many researchers, such as the authors of this paper, Tobii Pro Lab and the other commercial packages offer insufficient flexibility and insufficient control over the various analysis steps. Specifically, Tobii Pro Lab only visualizes a limited amount of the data available in a Glasses 2 recording (for instance, the gyroscope and accelerometer data which may be helpful to classify participant movement are not featured in the interface at all), is limited in functionality (for instance, only one fixation classifier can be chosen and its exact algorithm is not known given that the implementation’s source code cannot be inspected), is not extensible and provides a workflow for manual mapping gaze data to objects-of-interest that has been found to be inefficient (Benjamins et al., 2018).

In this paper, we present GlassesViewer, an open-source tool that enables easily navigating the study- and recording structure produced by the Tobii Pro Glasses 2 and provides tools for converting the recorded data to an easily readable format and synchronizing all data streams and videos together. It furthermore provides a graphical user interface that can visualize and replay all data streams and both scene and eye videos in a recording, contains an interface for manual or automatic event classification, as well as for viewing and adjusting the output of a manual mapping procedure. Via integration with GazeCode (Benjamins et al., 2018), users can manually map the events that were classified with GlassesViewer’s tools (e.g., fixations) to objects of interest in an intuitive and efficient manner, and then view these mapped events on the recording’s timeline in the GlassesViewer tool. In contrast to Tobii Pro Lab, the GlassesViewer software presented here has extensive built-in visualization capabilities, is built to be highly configurable and extensible and uses data input and output formats that allow for easy interoperability with other software tools, such as the featured integration with GazeCode. We consider GlassesViewer and GazeCode together to offer a free and open-source^1^ replacement for most of the functionality offered by the manufacturer software. For a feature comparison, please refer to Table 1. GlassesViewer is available from https://github.com/dcnieho/GlassesViewer and GazeCode from https://github.com/jsbenjamins/gazecode. GlassesViewer has been tested with recordings made with Tobii Pro Glasses 2 firmware versions 1.22.0-zucchinipaj, 1.25.0-citronkola and 1.25.3-citronkola.

**Table 1:** A comparison of the features offered by the GlassesViewer and GazeCode combination, and the Tobii Pro Lab Analysis module

Feature	GlassesViewer + GazeCode	Tobii Pro Lab Analysis module
Scene video playback	yes	yes
Eye video playback	yes	no
Data stream timeseries plots	horizontal and vertical eye orientation (per eye), pupil size, eye angular velocity, gyroscope, accelerometer	horizontal and vertical gaze position on the video (binocular only), gaze velocity
TTL signal visualization on timeline	yes	yes
Coding by selecting intervals on timeline	yes	no
Interactive event classifier parameter adjustments	yes	yes
Manual event mapping	yes, using coding buttons or keyboard keys	yes, using manually defined events coupled to keyboard keys
Supported event types for manual mapping	any	fixations
Automatic event mapping	no	yes, for 2D planes
AOI analyses	no	yes
Extensible event classifiers	yes	no
Video export with overlaid visualization	no	yes
Data export	yes, to MATLAB file	yes, to csv file

## The GlassesViewer tool

The GlassesViewer toolbox consists of tools for (1) selecting, (2) parsing and (3) viewing and analyzing Tobii Pro Glasses 2 recordings. Below we provide a detailed description of the toolbox’s functionality. For the reader who prefers to try out the tools directly, we however recommend starting by following the steps described in the quick start manual manual.md that is available in the GlassesViewer repository on GitHub. The first two tools described in the Sections “Recording selector” and “Recording parser” are building blocks for the glassesViewer graphical user interface described in the Section “Recording viewer and analyzer graphical user interface”. These first two tools can however also be used stand-alone.

### Recording selector

**Fig. 1:**
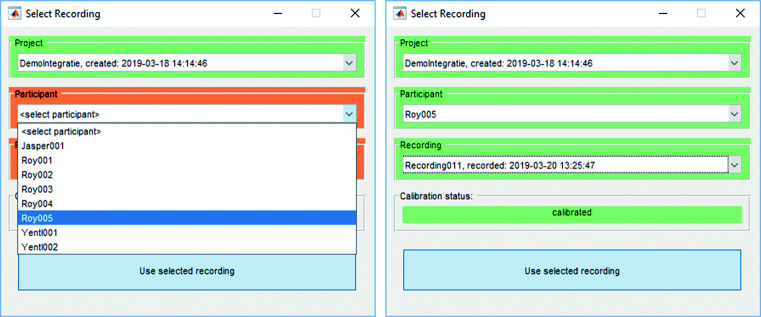
The recording selector interface. When provided with the directory structure created on the SD card by the Tobii Pro Glasses 2, this dialogue shows all the projects contained in the directory structure. For each of the projects, it shows all the participants from whom data was recorded (*left panel*), and for each participant all their recordings. Once a recording is selected, the dialogue shows whether the recording was preceded by a successful calibration (*right panel*)

The Tobii Pro Glasses 2 stores studies and recordings in a complex directory structure on its SD card. The names of studies and recordings in this directory structure are random strings (e.g. recording gzz7stc, which is part of project raoscyb), and as such not human-readable. This makes it hard to locate a specific study or recording. GlassesViewer therefore provides two functions to easily navigate this structure. First, the function G2ProjectParser determines what studies (e.g., DemoIntegratie) and recordings (e.g., Recording011) are contained in a directory and stores it in a Microsoft Excel file, lookup.xls. This file contains information about all the studies in the directory, and all the recordings in each study. Second, the function recordingSelector reads this lookup file and presents a graphical study and recording selection interface (Fig. 1). When a recording is selected, the tool provides the path to this recording in the directory structure, so the recording can be located.

### Recording parser

Each recording made by the Tobii Pro Glasses 2 is comprised of a scene video file, an optional eye video file, and a gzipped text file containing the headset’s data streams encoded in packets of json-formatted text data. To make the data in these files accessible, the GlassesViewer toolbox contains functionality to parse, organize and synchronize these data streams. It can furthermore extract the timestamp for each frame in the video streams, and then synchronize these frames to the recording’s data streams. Parsing of a recording is performed by the function getTobiiDataFromGlasses which provides the processed data streams and video frame timestamps to the caller in a MATLAB structure, and also stores this structure to a file livedata.mat in the recording’s directory. During parsing, getTobiiDataFromGlasses cleans up the data streams by checking if a set of conditions is met for each gaze sample—such as whether data for all gaze data streams is available for a given sample. Gaps sometimes occur in the gaze data recorded by the Glasses 2 and can complicate the analysis of eye-tracking data (Hessels et al., 2015). getTobiiDataFromGlasses detects these gaps and fills them with “missing” gaze samples in such a way that the recording’s gaze data sampling interval is preserved. getTobiiDataFromGlasses furthermore provides support for parsing recordings consisting of multiple segments, which occur when the Tobii Pro Glasses 2 splits recordings longer than approximately an hour.

### Recording viewer and analyzer graphical user interface

**Fig. 2:**
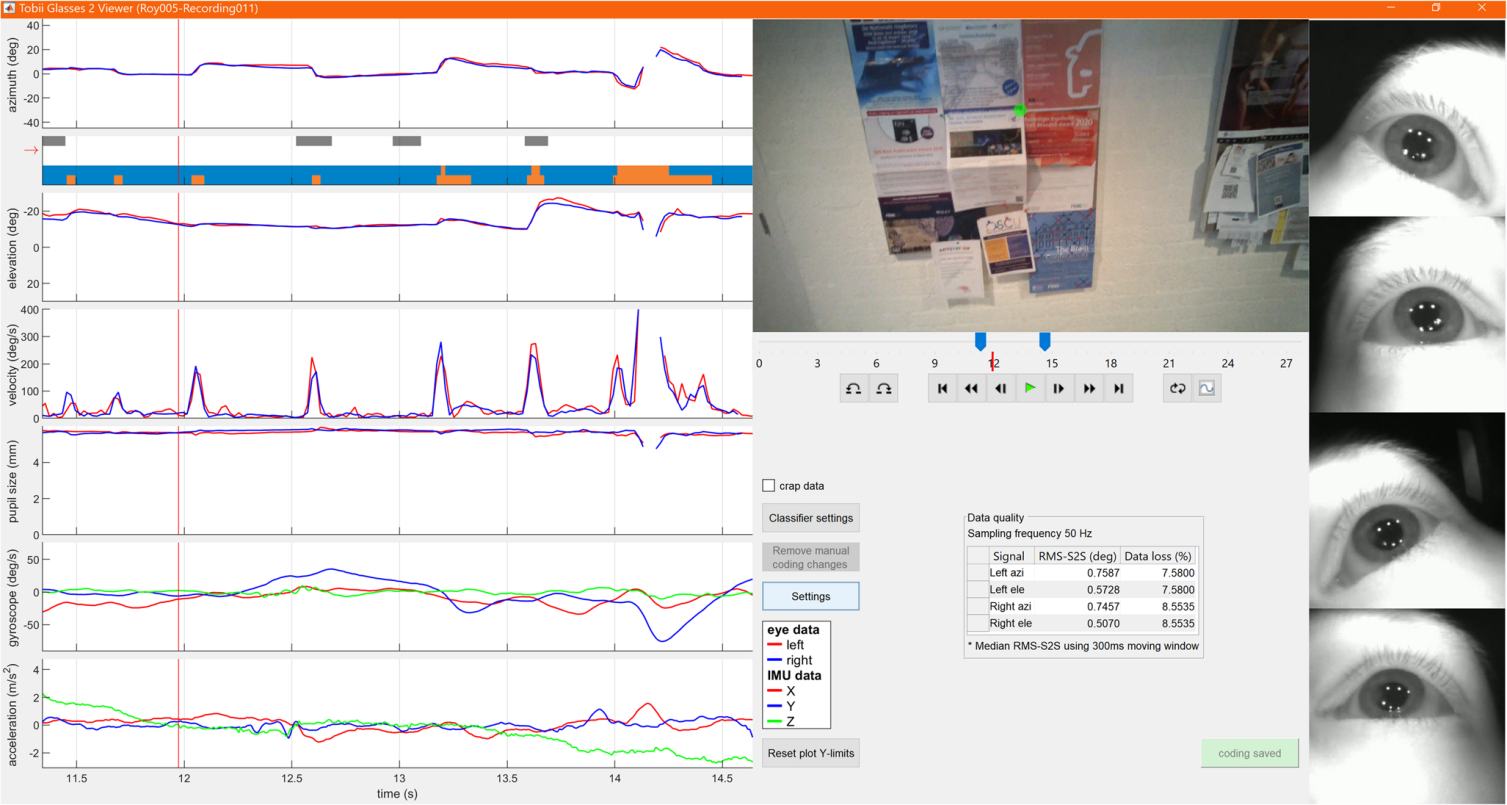
The GlassesViewer recording viewer. On the left of the interface, raw data streams are plotted along with a scarf plot showing multiple annotation streams. To the right of these data stream plots, the scene and eye videos are shown, along with a media player toolbar for playback of the recording, and to allow selecting the time window for which data are plotted in the data stream plots. Furthermore shown when a recording is first opened is a panel indicating the data quality (RMS-S2S precision and data loss) of the recording

The function glassesViewer selects and loads a recording using the functionality provided by the two tools described above and shows a flexible graphical user interface in which all data streams along with the video files can be viewed and annotated (see Fig. 2). glassesViewer furthermore supports loading annotations (denoting, e.g., fixation episodes or walking episodes) from text files, and the automatic generation of annotations through running built-in or user-provided event classifiers. The glassesViewer tool’s settings can be configured through a JSON file, or provided as a MATLAB struct when invoking the function. Example default settings are provided in the defaults.json file included with the glassesViewer tool. A complete manual for the glassesViewer interface as well as all the settings in the JSON file is found in the readme.md file included with the glassesViewer tool.

#### Viewing a recording

**Fig. 3:**
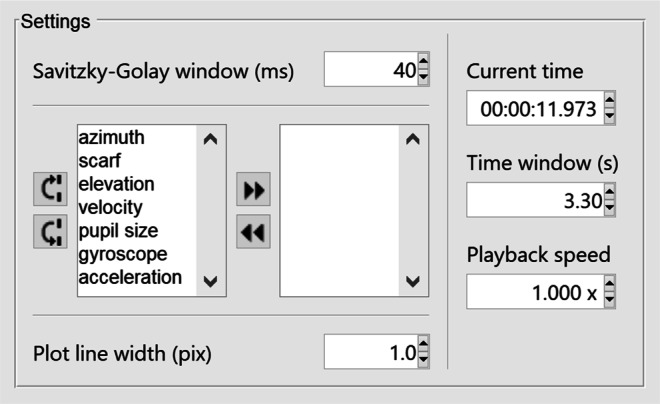
The settings panel of the glassesViewer recording viewer enables changing various settings while a recording is loaded in the glassesViewer interface. The Savitzky–Golay window setting changes the number of filter taps used for calculating instantaneous eye velocity, and the two list boxes underneath denote which time series are visible in the interface and in which order (*left box*). Data stream plots can be hidden from view by moving them to the right box, allowing maximum screen space for the data stream plots that the user is interested in viewing

Figure 2 shows a Tobii Pro Glasses 2 recording loaded in the glassesViewer interface. The left side of the interface shows a series of data streams plotting the raw gaze direction vectors decomposed into Fick angles (Fick, 1854; Haslwanter, 1995) and plotted as azimuthal and elevational eye orientations; the corresponding eye velocity as computed from the eye orientation time series; pupil size; and the raw gyroscope and accelerometer time series that are available in a recording. Furthermore shown is a scarf plot denoting manually or algorithmically created event streams that will be discussed in the Section “Manual and algorithmic event annotation” section below. Which of the data stream plots are shown is configurable, both in the settings JSON file, and in a settings panel in the glassesViewer interface itself (Fig. 3). The eye velocity is computed in the following manner. First, smoothed azimuthal (*𝜃*) and elevational (*φ*) eye velocity time series are computed using a Savitky–Golay differentiation filter (Savitzky & Golay, 1964). The eye velocity ($\dot {\omega }$) is then computed by $\dot {\omega }=\sqrt {\dot {\theta }^{2}\cos \limits ^{2}\varphi +\dot {\varphi }^{2}}$ (Niehorster et al., 2015).

**Fig. 4:**
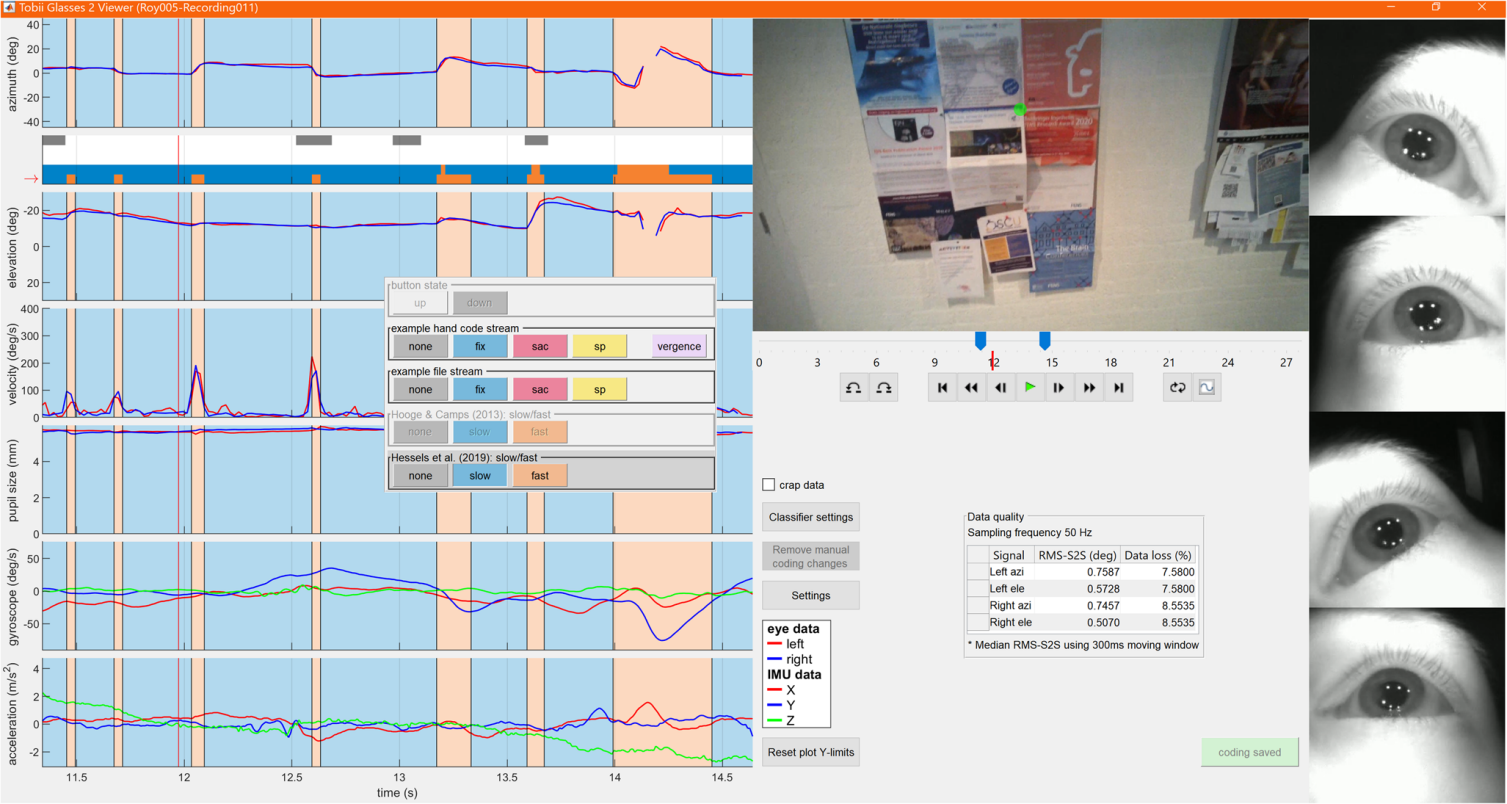
The glassesViewer recording viewer showing an event coding. When an event stream containing coding for the current time window is selected, the coded episodes are shaded on each of the data stream plots. Furthermore shown is an open coding panel allowing one to change an already coded episode to another label, or to place a new code in each of the event streams. The currently active event stream is indicated by the *red arrow* next to the scarf plot

To the right of the data stream plots, the scene video and, if available, eye videos are shown. Underneath the scene video, a media player toolbar is provided, allowing to play and pause a recording, as well as jump forward and backward in time, adjust the time window that is shown in the data stream plots, and loop the playback during that time window. On top of the scene video, the recorded gaze position is shown as a green dot when the Glasses 2 headset based its reported gaze position on data from both eyes, and as a red dot when the reported gaze position is based on data from a single eye.^2^

Underneath the media player toolbar, a panel indicating the data quality of the recording is shown. To ensure that a recording’s data quality is sufficient to allow for a valid analysis, it is of utmost importance that the user is aware of these statistics (Holmqvist et al., 2012; Niehorster et al., in press; Nyström et al., 2013; Hessels et al., 2015; Niehorster et al., 2018). We have therefore decided to display them prominently in the interface. Specifically, two aspects of data quality are reported, separately for the azimuth and elevation gaze direction signals of the left and right eyes. First, a measure of the random variation in the gaze direction signal (often referred to as its precision) is provided by means of the root-mean-square of the distance between the gaze directions of adjacent gaze samples (RMS-S2S). The RMS-S2S measure was calculated with a moving window (default length 300 ms), yielding a list of RMS-S2S values. To get an estimate of the RMS-S2S during periods where the eyes move slowly (fixations on static or moving objects while the participant’s head is also either static or moving), the median of all the RMS-S2S values was computed and displayed in the interface. This RMS-S2S measure is intended to provide a value that can be used to compare various recordings performed with the same system setup, making the user aware of recordings that are of significantly worse quality than others in their set. Second, data loss, the number of invalid gaze samples as a percentage of all gaze samples in the recording is presented.

#### Manual and algorithmic event annotation

The glassesViewer interface provides annotation functionality, allowing users to manually label episodes in the recording, or to have episodes labeled algorithmically. For this functionality, glassesViewer uses event streams. Each event stream is a separate, user-configured contiguous series of labels denoting specific episodes in the recording. Users could for instance in one stream code whether the eyes are rotating slowly (slow phase; ‘fixation’ or ‘smooth pursuit’) or rapidly (fast phase; ‘saccade’) in the head of the participant (Hessels et al., 2018), while in another stream coding whether the participant is walking or standing still and use a third stream to view a coding of which object-of-interest the participant is looking at. Event streams and their annotation categories are set up in the JSON settings file, which is documented in the readme.md file. The produced coding is stored in a file coding.mat alongside a recording’s livedata.mat file.

At any time, one of the coding streams is active, meaning that its codes are displayed through highlighting in each of the data stream plots (see Fig. 4). By clicking on any of the data stream plots, a new annotation can be added ranging from the end of the previous annotation until the clicked time. When clicking an existing annotation, the labeled category can be changed or the annotation can be removed. The start- and end time of annotations can be changed by dragging and annotations can be split by holding down the shift key while clicking on the time where a new annotation should be inserted. Lastly, users can set up their annotation scheme such that flags can be applied to them. For instance, when coding for fixations and saccades, users can set up a flag to augment the saccade annotation with information about whether the saccade has a vergence component or not.

**Fig. 5:**
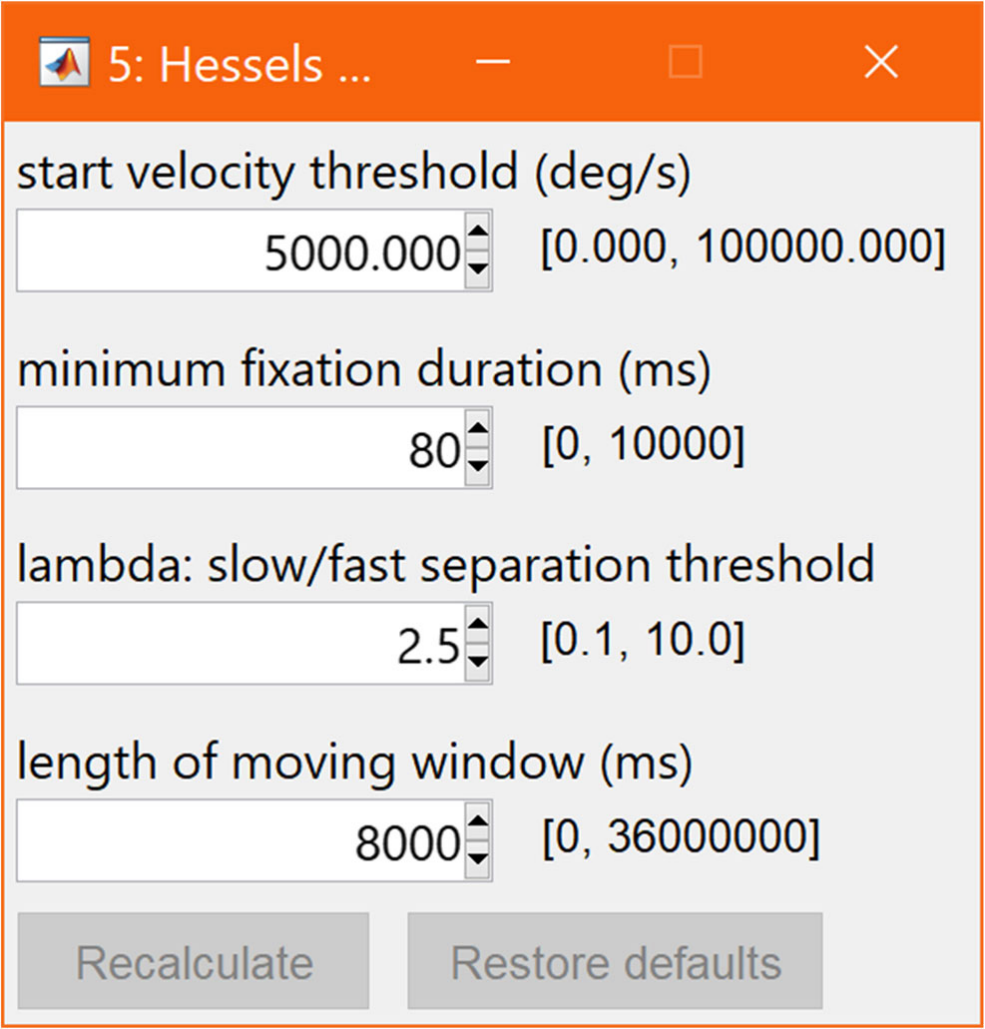
Classifier settings for the glassesViewer recording viewer. Shown are the settings for the Hessels et al., (in press) slow and fast phase classifier. For each setting, a textbox is available through which the value can be changed, and besides the textbox, the range of allowed values is indicated. The recalculate button performs event classification with the selected settings, and the restore defaults button resets the parameter values to their initial values as defined in the JSON settings file

Besides manually produced annotations, event streams can also come from multiple other sources:

***TTL signals:*** The Tobii Pro Glasses 2 includes a TTL port through which events, such as sync signals or button presses, can be timestamped and recorded. glassesViewer can be set up such that an event stream is automatically generated from the TTL activity.
***Text files:*** To provide simple interoperability with other tools, an event stream can be loaded from a text file. If the user manually adjusts the loaded annotations, they can be reset to those contained in the text file with a button in the interface. A full description of how to set this up is provided in the readme.md file that comes with the GlassesViewer toolbox.
***Classification algorithms:*** Lastly, glassesViewer can produce annotations by calling user-provided algorithms. These algorithms are provided with a recording’s data streams and are expected to return an event stream. The MATLAB function to call is defined in the JSON settings file, along with parameter settings. These parameters can be marked as user-settable, in which case a dialogue becomes available in the interface (Fig. 5) allowing the user to change the parameter settings of the algorithm and rerun it to produce an updated event stream. glassesViewer comes with two classifiers (Hooge and Camps, 2013; Hessels et al., in press) that split the gaze data into episodes where the eye moves fast, and episodes where the eye moves slowly. Shown in Fig. 4 is the classification produced by the Hessels et al. (in press) algorithm, and in Fig. 5 the settings for this algorithm are shown.

Regarding the use of event classification, it is important to realize that use of the event classification algorithms provided by GlassesViewer comes with the same caveats as use of those provided by Tobii Pro Lab or any other software package. Specifically, the user has to assure themselves that the algorithm and its settings produce classifications (e.g., “fixations”) that have the properties desired for further analysis. Example properties that may be relevant for specific use cases are that all events are at least of a specific minimum duration, that eye velocity during a fixation episode does not exceed a given threshold, or conversely that the slow eye-in-head gaze shifts associated with pursuit or VOR activity do not break up the classified event. These are properties of the event classifier output that have to be determined a-priori by the researcher (see, e.g., Hessels et al.,, 2017) and have to be checked by the researchers through inspection of the classified events using, e.g., GlassesViewer’s timeline (see Fig. 4). Failure to do either of these steps and unthinkingly accepting a default algorithm or its default settings may lead to improper event classifications and thereby invalid study conclusions.

## Integration with GazeCode

To provide researchers with a complete, more flexible and fully transparent replacement to Tobii’s software for viewing and analyzing recordings made with the Tobii Pro Glasses 2, a close but optional integration between the GlassesViewer toolbox and GazeCode (Benjamins et al., 2018) has been developed. GazeCode,^3^ available from https://github.com/jsbenjamins/gazecode, is a tool developed for efficient manual mapping of participant gaze onto the visual stimulus by assigning each look to a predefined category (such as “teapot”, “cup”, “spoon”). GazeCode was designed with a minimalistic interface that only shows the information necessary for this task and allows for straightforward keyboard-based operation. As such, a test performed by Benjamins et al., (2018) found that coders were able to annotate the same gaze data with GazeCode at double the rate they obtained when using the Tobii Pro Lab software.

By means of the integration between GazeCode and GlassesViewer, GazeCode can now use GlassesViewer’s functionality to parse and load a Tobii Pro Glasses 2 recording. It can furthermore load any of the event coding streams produced with the GlassesViewer tool, or invoke GlassesViewer’s functionality to produce these event streams automatically. Finally, after the researcher is done manually mapping, e.g., a participant’s gaze to objects in the world, the new event stream containing the researcher’s annotations is stored in the coding.mat file, allowing it to be viewed on the timeline provided by the glassesViewer interface and potentially manually adjusted.

## Example use and workflow

To demonstrate the workflow using the GlassesViewer and GazeCode tools, we have made a brief example recording where a participant was asked to search a notice board for red pushpins used to attach posters to the board, among pushpins with different colors. The participant was instructed to press a button on a custom-built response box connected to the Glasses 2’s recording unit (further described in Appendix) for each red push pin that they found. When loading the example recording from the demo_data directory included with the glassesViewer tool, these button presses are visualized as an event stream, and they can be chosen in GazeCode as the stream for which to conduct manual mapping.

The complete example workflow including all steps one may wish to perform in the glassesViewer interface, further manual mapping in GazeCode and then reviewing this mapping in glassesViewer is provided in the manual.md file that is included with the glassesViewer tool. The user is referred to this manual for an up-to-date walk-through of the glassesViewer tool.

## Conclusions

In this article, we presented GlassesViewer, a toolbox for viewing and analyzing recordings made with the Tobii Pro Glasses 2 head-mounted eye tracker. It provides tools for selecting, parsing, viewing and manually or automatically annotating such recordings. It is a flexible and fully transparent tool which, together with the efficient GazeCode manual mapper, provides a replacement for most of the functionality offered by the analysis module of the commercial Tobii Pro Lab software.

## Figures and Tables

**Fig. 6:**
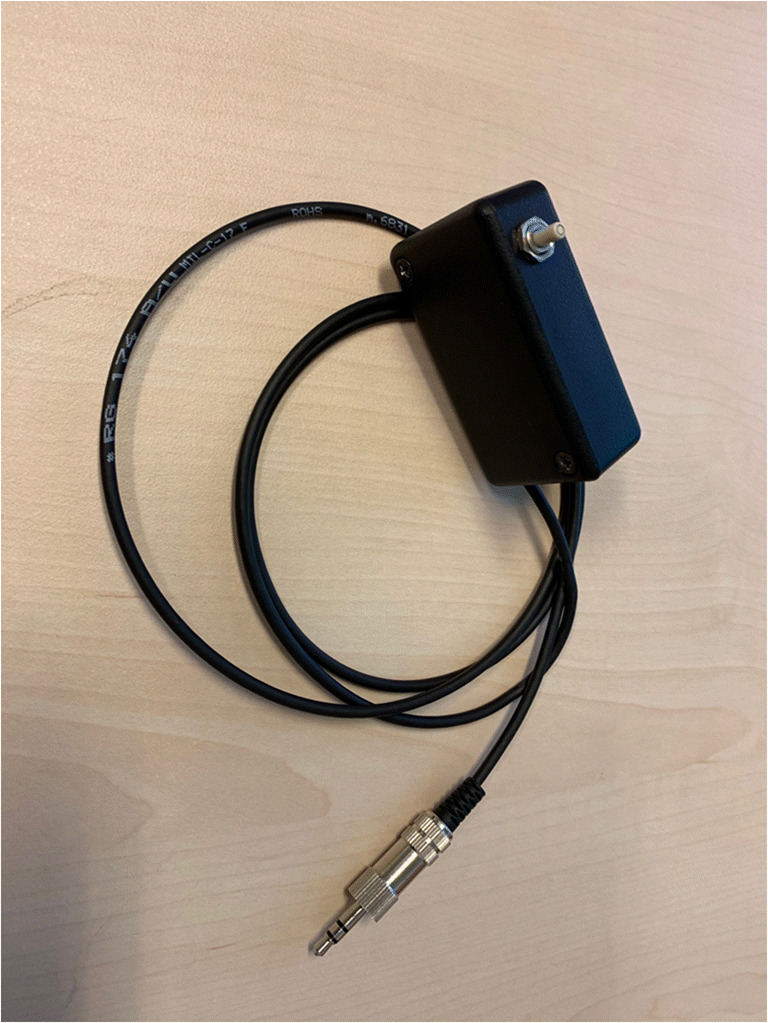
Button box for Tobii Pro Glasses 2. As long as the button is pressed, this box sends a TTL signal over a 3.5 mm jack that can be plugged directly into the sync port on the Tobii Pro Glasses 2 recording unit

## Appendix: A button box for use with the Tobii Pro Glasses 2

A button box (see Fig. 6) was built by our technician at Utrecht University. It is a one-button box that sends a TTL signal as long as the button is pressed. It operates on one CR2032 battery, and can be plugged directly into the Tobii Pro Glasses 2 recording unit. It is possible that similar products could be bought off-the-shelf.

## Footnotes

1 Note however that both require MATLAB to run, which is not open-source nor free software.

2 The Tobii Pro Glasses 2 determines the gaze position in the scene video from binocular data. If data for only one eye is available, gaze position on the video is determined under the assumption that the vergence distance of the two eyes did not change since binocular data was last available. In some experimental situations this is a valid assumption, but not in all. As such, we color-code the gaze marker in the video to make the researcher aware that they must decide whether to use gaze position data on the video originating from only a single eye. For a note of caution regarding using vergence in pupil-based eye trackers, see Hooge et al., (2019).

3 Note that besides data from the Tobii Pro Glasses 2, GazeCode also supports data from SMI, Pupil-Labs and Positive Science head-worn eye trackers.
